# When and how to use Q methodology to understand perspectives in conservation research

**DOI:** 10.1111/cobi.13123

**Published:** 2018-07-20

**Authors:** Aiora Zabala, Chris Sandbrook, Nibedita Mukherjee

**Affiliations:** ^1^ Cambridge Centre for Environment, Energy and Natural Resource Governance, Department of Land Economy, University of Cambridge The David Attenborough Building Pembroke Street Cambridge CB2 3QZ U.K.; ^2^ Department of Geography, University of Cambridge Downing Place Cambridge CB2 3EN U.K.; ^3^ UN Environment World Conservation Monitoring Centre 219 Huntingdon Road Cambridge CB3 0DL U.K.; ^4^ Centre for Ecology and Conservation, College of Life and Environmental Sciences University of Exeter Cornwall Campus, Penryn Cornwall TR10 9FE U.K.

**Keywords:** biodiversity conservation, conflict management, conservation policy, decision‐making, governance, human perspectives, social research, values, conservación de la biodiversidad, gobernanza, investigación social, manejo de conflictos, perspectivas humanas, políticas de conservación, toma de decisiones, valores, 生物多样性保护, 冲突管理, 保护政策, 决策, 治理, 人们的观点, 价值观, 社会研究

## Abstract

Understanding human perspectives is critical in a range of conservation contexts, for example, in overcoming conflicts or developing projects that are acceptable to relevant stakeholders. The Q methodology is a unique semiquantitative technique used to explore human perspectives. It has been applied for decades in other disciplines and recently gained traction in conservation. This paper helps researchers assess when Q is useful for a given conservation question and what its use involves. To do so, we explained the steps necessary to conduct a Q study, from the research design to the interpretation of results. We provided recommendations to minimize biases in conducting a Q study, which can affect mostly when designing the study and collecting the data. We conducted a structured literature review of 52 studies to examine in what empirical conservation contexts Q has been used. Most studies were subnational or national cases, but some also address multinational or global questions. We found that Q has been applied to 4 broad types of conservation goals: addressing conflict, devising management alternatives, understanding policy acceptability, and critically reflecting on the values that implicitly influence research and practice. Through these applications, researchers found hidden views, understood opinions in depth and discovered points of consensus that facilitated unlocking difficult disagreements. The Q methodology has a clear procedure but is also flexible, allowing researchers explore long‐term views, or views about items other than statements, such as landscape images. We also found some inconsistencies in applying and, mainly, in reporting Q studies, whereby it was not possible to fully understand how the research was conducted or why some atypical research decisions had been taken in some studies. Accordingly, we suggest a reporting checklist.

## Abstract (es)

Cuándo y Cómo Usar la Metodología Q para Entender Perspectivas en Investigación sobre Conservación

**Resumen**

Entender las perspectivas humanas es de suma importancia en una gama de contextos de conservación, por ejemplo, en la superación de conflictos o en el desarrollo de proyectos que sean aceptables para los actores relevantes. La metodología Q es una técnica semi‐cuantitativa única que se usa para explorar las perspectivas humanas. Se ha aplicado durante décadas en otras disciplinas y recientemente ha ganado terreno dentro de la conservación. Este artículo ayuda a los investigadores a evaluar cuándo es útil la Q para una pregunta dada sobre conservación y lo que su uso involucra. Para esto, explicamos los pasos necesarios para realizar un estudio Q, desde el diseño de la investigación hasta la interpretación de los resultados. Proporcionamos recomendaciones para minimizar sesgos en la realización de un estudio Q los cuales pueden influir mayormente en el diseño del estudio y la recolección de datos. Llevamos a cabo una revisión estructurada de la literatura para examinar en qué tipo de contexto empírico de conservación se ha utilizado la Q. Identificamos 52 estudios. La mayoría fueron casos nacionales o sub‐nacionales, pero algunos también respondieron a preguntas multinacionales o globales. Encontramos que la metodología Q se ha aplicado a cuatro tipos generales de objetivos de conservación: manejar conflictos, diseñar alternativas de gestión, entender la aceptabilidad de políticas, y reflexionar críticamente sobre los valores que influyen implícitamente en la investigación y la práctica. Por medio de estas aplicaciones los investigadores han encontrado puntos de vista inadvertidos, han entendido opiniones en profundidad y han descubierto puntos de consenso que facilitaron la resolución de desacuerdos complicados. La metodología Q tiene un procedimiento claro, pero también es flexible, lo que permite a los investigadores explorar puntos de vista a largo plazo, o puntos de vista sobre objetos diferentes de oraciones, como imágenes de paisajes. También encontramos inconsistencias en la aplicación y, principalmente, en el reporte estudios Q, por los cuales no fue posible entender completamente cómo se realizó el estudio o por qué se tomaron algunas decisiones de investigación atípicas en algunos estudios. De acuerdo con esto, sugerimos una lista de cotejo para los reportes.

## 摘要

理解人们的观点在许多保护的情境中都十分重要, 如解决冲突或开发能让利益相关者接受的项目。 Q 方法是一个独特的用于探究人们观点的半定量方法。它在其它学科中已有数十年的应用, 近来也被用于保护领域。本文帮助研究者评估 Q 方法何时可以用于给定的保护问题, 以及它的使用涉及到什么。为此, 我们解释了进行 Q 研究从研究设计到结果解读的必要步骤。我们提供了减少 Q 研究中研究者误差的建议, 其影响主要发生在实验设计和数据收集阶段。我们还进行了结构化文献综述来分析 Q 方法在哪类实证保护研究中得到应用。我们找出了 52 个研究, 大部分是国家以下或国家水平的案例, 但也有些涉及多国家或全球水平的问题。我们发现, Q 方法目前用于 4 大类保护目标:处理冲突、设计管理替代方案、分析政策可接受性, 以及批判地思考暗中影响研究和实践的价值观。通过这些应用, 研究者可以发现隐含的意见, 深度理解人们的观点, 并找到有助于解决分歧的共识点。 Q 方法有明确的操作步骤, 但也有一定灵活性, 可以让研究者探索长远的观点, 或是人们对事物 (如景观图像) 而非语句的看法。我们还发现 Q 方法在应用中存在不一致, 主要是对 Q 研究的报告, 据此我们难以完全理解研究是如何进行的, 以及一些研究中为何做出了非典型的研究决定。因此, 我们建议使用一份报告检查表。**【翻译: 胡怡思; 审校:魏辅文】**

## Introduction

It is widely recognized that conservation is a social endeavor, meaning that social research approaches have a critical role to play in conservation science (Teel et al. 2018). In multiple contexts, there is a need to understand the different values and views of individuals with respect to issues important for conservation. For example, the success and failure of conservation interventions can hinge strongly on whether and how the views of different stakeholders are understood and integrated (Redpath et al. 2013; Madden & McQuinn 2014; Bennett 2016) and the extent to which proposed solutions are perceived as acceptable (e.g., Chandran et al. 2015). There is also value in understanding different perspectives within conservation, which can foster critical self‐reflection among conservationists about objectives and approaches (e.g., Holmes et al. 2016).

The Q methodology (hereafter Q) is exploratory and semiquantitative, and provides a clear and structured way to elicit stakeholder views (termed “operant subjectivities*”* in the Q literature) on an issue. It categorizes these individual viewpoints into clusters of value positions, belief systems, or mental models (McKeown & Thomas 2013). Issues might include human–nature conflicts, management of a threatened species, or internal debates about strategy within a conservation organization. Opinions around each conservation issue can be very diverse. In order to address these issues successfully, it is often important to take different opinions into account. Researchers can use Q to uncover the diversity of views regardless of whether these views are frequent within a population (Watts & Stenner 2012).

Of fundamental importance to Q is that it combines quantitative and qualitative data and analytical techniques. This is an important strength of the approach, but can also lead to confusion. In our experience, conservation researchers (including authors of this paper) who have a background in positivist and quantitative research can be attracted to Q initially because it has familiar features (such as multivariate data reduction techniques) that are not present in other purely qualitative methods. However, it is important for anyone considering the use of Q to recognize that, as a methodology, it is based on philosophical and epistemological premises that are non‐positivist (Watts & Stenner 2005; these concepts are introduced in Moon and Blackman [2014]). Following a non‐positivist approach, in Q the researcher is not considered to be a neutral actor revealing truth. Rather, they play an active role in shaping the analysis and the interpretation of the results, grounded in their knowledge of the study system. As discussed further below, Q engages researchers’ intuition and creativity (as well as their quantitative analytical skills) and allows them to play an active role throughout the process.

We identified four primary differences between Q and other social research methods used for similar purposes. First, it provides numerical results to support the perspectives interpreted and therefore combines benefits of quantitative and qualitative approaches. Second, it uncovers how different but related topics are interconnected by requiring respondents to consider such topics simultaneously (unlike standard surveys, which elicit opinions about each topic separately). Third, in order to synthesize perspectives into a manageable set, Q focuses on similarities between individuals (as opposed to similarities between questions or variables, i.e., a so‐called inverted factor analysis [FA]). Finally, it can mitigate certain response biases because respondents are required to engage explicitly with opinions that they might deem inappropriate or unexpected.

The Q methodology can be fruitfully combined with other methods, such as interviews (Rastogi et al. 2013) or surveys (Hagan & Williams 2016). However, most often it is used as a standalone technique. In comparison with surveys, Q yields more nuanced and sophisticated opinions (Kamal et al. 2014). It offers a middle ground between the structure of surveys and the depth of interviews, and combines advantages of both. It is most frequently administered with individuals, and in such cases it is relatively free of certain psychological biases such as dominance effect (Mukherjee et al. 2015), which can affect methods administered in groups (e.g., focus group discussions).

Conversely, because the sampling of respondents is usually not random, results from Q cannot be readily extrapolated to wider populations. Also, Q arguably leaves less freedom of interpretation than qualitative discourse analysis and interviews because perspectives in Q are limited to a set of items presented to respondents and, to some extent, to the quantitative results.

The methodology originated with Stephenson's (1935) proposal to apply FA to find similarities among people (based on characteristics such as attitudes and behavior). Although its origins are in psychology, Q was later adopted more widely in political science (Watts & Stenner 2012) and subsequently in human geography (Eden et al. 2005), healthcare (Valenta & Wigger 1997; Cross 2005), and other fields. In conservation, however, it has had limited application relative to other techniques used for similar purposes, such as interviews and focus group discussions. Because understanding perspectives is at the heart of a wide range of conservation questions and problems, this technique has significant potential. Currently, there are neither best practice guidelines for Q in conservation nor reviews of its application.

We discussed the potential of using Q in conservation research, based on a structured review of 52 empirical studies. We sought to provide a first‐stop reference to assess the usefulness of the methodology for a given conservation question. We explain how to conduct a Q study and outline required resources. We assessed in which conservation contexts and the types of questions that have been addressed using Q and provided recommendations for its application.

## How to Conduct a Q Study

**Figure 1:**
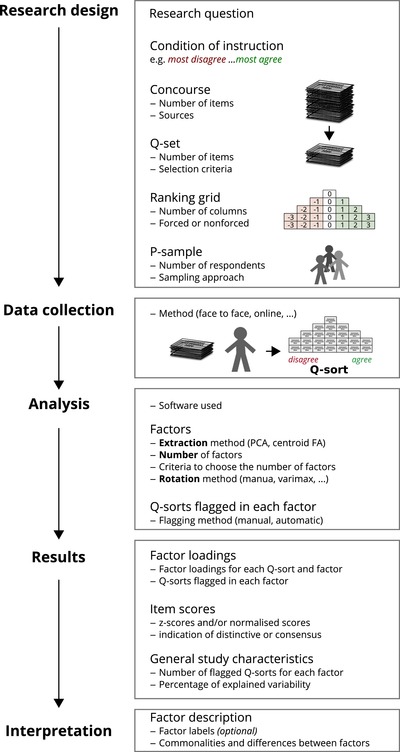
Research process of Q methodology (concourse, unfiltered set of items about the topic; Q‐set, set of items to rank; P‐sample, sample of respondents; Q‐sort, ranking of items by a single respondent; PCA, principal components analysis; FA, factor analysis). We suggest that the elements with dashes be used as a guideline for standard reporting.

A Q study can be divided in 4 stages (Fig. 1): research design, data collection, analysis, and interpretation. These steps may be iterative, for example, a preliminary interpretation of results may trigger reconsidering the number of perspectives to interpret. However, we describe them linearly for ease of understanding. More detailed explanations of the procedure are in Watts and Stenner (2012), McKeown and Thomas (2013), and, with a focus on environmental research, Webler et al. (2009). A collection of worked examples is in Van Exel and de Graaf (2005).

### Stage 1 Research Design

The first step is to identify the topic that sets the scope for the study and the overall question to ask respondents (e.g., “Sort these opinions from most like what I think to least like what I think”). The next step is to collect a comprehensive list of items (statements) that suggest a subjective opinion about the research topic. The comprehensive list is a proxy for the ‘concourse’: the full spectrum of items reflecting all possible opinions around the topic of concern (which in theory would be infinite). Items may be drawn from several sources, such as written material (newspapers, scientific publications, etc.), interviews or expert consultation. It is important to document this process.

From the concourse, researchers select a representative sample of items (the Q‐set), which respondents will rank. Approaches to selecting this sample of items vary. For example, items may be categorized according to subtopics and a balanced number from all subtopics may be chosen (e.g., Jacobsen & Linnell 2016).

The aim of Q is to uncover the diversity of opinions, irrespective of whether they are predominant in the population. Consequently, the sample of respondents (the P‐sample) is usually a nonrandom selection of individuals, and the sampling strategy is primarily purposive (selected following criteria other than randomness). Ideally, for the purposive sampling, researchers would be broadly familiar with the stakeholders and their views beforehand, which facilitates selecting respondents with a variety of opinions. In some cases, snowball or convenience sampling is used (whereby respondents are chosen if a previous respondent mentions them or if they are easily available [Saumure & Given 2012]). Alternatively, respondents can be selected based on observable characteristics, such as socioeconomic or professional status. Because of the use of inverted FA, an unusual feature of Q is that the rule of thumb that larger sample sizes are better does not necessarily apply. Powerful Q results can be obtained with very small samples (e.g., Sandbrook et al. 2013).

### Stage 2 Data Collection

Respondents are asked to rank the items and this ranking is called Q‐sort. They rank the items over a grid with columns. This grid usually represents a simplified bell‐shaped distribution (an example of such a grid is the triangular grid in the part of data collection’ in Fig. 1), the kurtosis of which varies across studies (i.e., the proportion between width and height). Respondents follow a condition of instruction for the ranking (e.g., from most agreement to most disagreement). The instruction can refer, for example, to agreement with items, importance, acceptability, or closeness to respondent's beliefs. Items placed in the same column receive the same ranking score. Researchers choose whether they ask respondents to fit all items in the slots within the grid or to allow, in each column, more (or fewer) items than the predefined slots (forced and nonforced distribution, respectively). The process is usually face to face, but it can also be by post or online (using software such as Q‐assessor, FlashQ, Q‐sortware, or WebQ). It is common to collect qualitative data as part of the Q process, either through respondents articulating their thought process during the ranking or after they have completed the process. Commonly, respondents are asked to explain the rationale of ranking items in the most extreme columns. Qualitative data thus collected play an important role in the interpretation.

A Q‐sort represents the perspective of a single respondent, whereby the respondent expresses what items prompt their strongest subjective reactions (those ranked in the extreme columns) relative to other items. Respondents need to provide sufficient variability in their response, such that they reveal nuanced degrees of engagement with items. For example, responses suggesting they agree very much with half of the items and disagree with the rest must be avoided. A good strategy to ensure this variability is to use a grid with a sufficiently ample range of columns. In the example in Fig. 1, this range takes all integers from ‐3 to 3, for instance. If nonforced distribution is preferred, the data collector should ensure that respondents do not cluster items in just a few columns and remind them that items should be ranked relative to one another rather than independently.

Piloting is an important stage in Q to ensure that statements are comprehensible for the respondents and to identify other unforeseen problems. Lessons learned from piloting can be used to revise the Q‐set or make adjustments to the Q‐sort process.

### Stage 3 Analysis

All the Q‐sorts collected are compared and grouped by similarity. Each group is then summarized as a single perspective (full analytical process is explained in Brown [1980]). This comparison, grouping, and summarizing is done through multivariate data‐reduction techniques (such as principal components analysis [PCA] or FA). There are a number of dedicated software packages available to analyze Q data (e.g., PQMethod [Schmolck 2014] and qmethod for R [Zabala 2014]).

As in standard PCA and FA, the data are reduced to a few factors (or components, i.e., the perspective shared by each group). This reduction is done in two main steps: extraction and rotation. The main analytical decisions in Q are as follows: the number of groups (i.e., number of factors), the method to extract factors (PCA or centroid FA), and the method to rotate factors (further analytical decisions discussed in Zabala and Pascual [2016]).

Extracting the factors consists of summarizing all individual responses into a few representative responses. The criteria used to choose the number of factors to extract vary across studies (details in Watts and Stenner [2012]). Most studies consider two or more criteria. In addition to typical criteria used in conventional FA or PCA, in Q a preliminary interpretation of the resulting perspectives can also be used to decide the number of factors. For example, if a perspective does not seem realistic or is qualitatively similar to others, the researcher may extract fewer factors.

Next, rotating the factors is akin to changing the viewpoint from where results are observed, much like changing the range of a scale or applying a logarithm. This is done to obtain a clearer and more interpretable structure of the results (further information on rotation in Brown [1980]). Other features of the analysis that are specific to Q are the percentage of the data variability explained by the results and the flagging of responses (i.e., tagging them as most representative of a given factor, both explained in detail in Watts and Stenner [2012]).

Two key results describe each shared perspective (factor). One set of coefficients, the factor loadings, indicate the relation between each respondent and factor. Factor loadings can be interpreted similar to the way correlation coefficients are interpreted. Another set of values indicate the relation between each item and factor. These values can be either *z* scores or normalized scores (Fig. 1). The *z* scores are the weighted average of the scores that similar respondents gave to an item. The normalized scores are integer approximations of *z* scores (but not the rounded values [Zabala 2014]). These item scores indicate how a hypothetical person representing a group of similar respondents (the factor) would rank the items.

The *z* scores give more precision about how strongly engaged each perspective is with each item. They are also used to determine whether an item is a consensus (with similar *z* scores across factors) and whether the item distinguishes a factor (significantly different *z* score in a factor compared with the rest).

### Stage 4 Interpretation

The interpretation of factors is based on a combination of the item scores, qualitative data (e.g., collected from respondents during the Q‐sort), and the researcher's understanding of the case and of respondent views. Both the *z* scores and the normalized scores indicate the ranking of items of a shared perspective. Items that are both distinctive and with the highest or lowest scores tend to be most useful for interpretation. When these items are identified, the interpreter evaluates why they are in that position. Consensus items indicate the common ground among all factors.

Each factor is usually given a meaningful label and described in detail. Although labels are not essential, they provide readers with a shorthand identification of what the perspective is about. Labels usually refer to the most distinguishing characteristic of the perspective, for example: “environmental stewards,” “production maximizers,” and “networking entrepreneurs” (Brodt et al. 2004). Labels can also be phrases, such as “separation of science and conservation” (e.g., Cairns 2012) or full sentences (e.g., Gruber 2011).

Studies using Q yield a clear output: a set of synthesized, shared perspectives. A detailed exposition of their differences and commonalities is usually discussed relative to subtopics of the research question. Disagreements may be as researchers expected but are sometimes surprising. These include cases in which the actual differences were less contentious than what was anticipated prior to the study (e.g., Visser et al. 2007; Mazur & Asah 2013). In some cases, general values were agreed but disagreement lay in the specifics of how to achieve shared goals (e.g., Cavanagh et al. 2016), or it was shown that further scientific research would not solve the existing social conflict (e.g., Neff & Larson 2014).

### Considerations for Applying Q

There are some fundamental considerations when applying Q that researchers should be aware of. First, as with many social research techniques, it is important to consider the risk of creating bias through respondent–interviewer interactions. A particular challenge occurs when respondents ask for clarification of the meaning of key terms in the statements, given that the interpretation of these terms can be a relevant point of difference between perspectives. In such cases, researcher answers should be consistent and avoid leading respondents toward a particular view. Second, respondents can find the sorting process challenging. Some take a long time to make decisions (there may be long periods of silence while respondents cognitively process items and decide), and others can find the need to sort items relative to each other frustrating or confusing. In both cases, the researcher needs to be patient and supportive of the respondent. Third, the choice of items influences the research critically: how respondents understand the message, whether the perspectives cover the topic sufficiently, and whether results are easily interpretable. Because the selection of items can be heavily influenced by the researcher, some studies report researcher bias as a potential problem (Ockwell 2008; Kamal et al. 2014). Critical to minimizing this risk is to make the item selection process systematic, exhaustive, and transparent. It is also important to keep alert to the possibility that qualitative data collected during Q‐sorts reveal a relevant point of view absent from the Q‐set. Fourth, in selecting items, there is a trade‐off between inclusiveness and cognitive overloading of respondents. Not all possible viewpoints can be captured by a limited set of items (Bredin et al. 2015*a*). Yet researchers need to delineate a set of items that respondents can sort in a reasonable time, otherwise the effectiveness and consistency of responses may be questioned (Swaffield & Fairweather 1996). Finally, the nonrandom sampling approach means that results cannot be directly extrapolated to a wider population and that frequencies cannot be estimated. Other methods applied during follow‐up studies can be used to explore the prevalence of positions identified using Q in the wider population (Danielson 2009).

Overall, conducting a Q study is not easy. The various stages in the process take considerable time, and in our experience some researchers underestimate its complexity due to the relatively straightforward quantitative analysis involved.

## Study Characteristics and Types of Questions of Q Studies in Conservation

To explore in which conservation contexts Q has been applied, we conducted a structured three‐pronged search of the literature. This search yielded 52 articles (sampling approach and articles reviewed are in Supporting Information).

**Figure 2:**
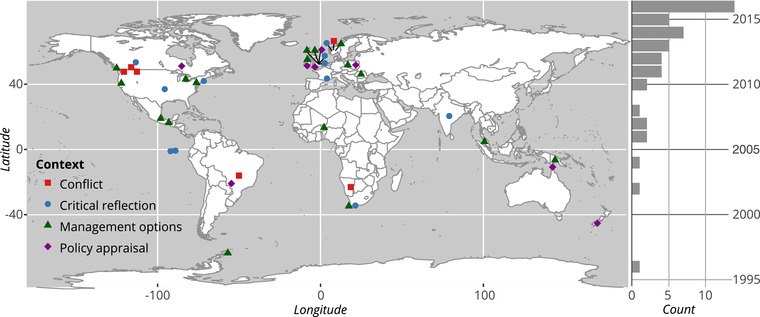
Location, context, and number over time of conservation‐related studies in which Q methodology was used (each symbol represents a study; context, decision context in which the study was conducted). Multicountry and global studies are not represented in the map. Studies published in 2017 are not shown on the graph.

Most empirical conservation studies in which Q was used were conducted in Europe and North America (Fig. 2). Africa has seen the fewest applications (we excluded studies written in languages other than English, which could partially explain the geographical clustering). The scale of application was predominantly at the subnational (*n* = 38) or national (*n* = 9) levels, the latter category included multisite studies in a single country. Fewer studies spanned countries or had global scope.

**Figure 3:**
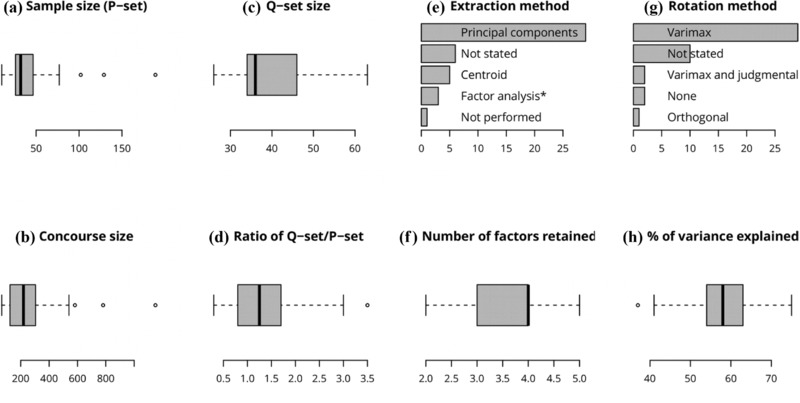
Characteristics of the research design (a–d) and analysis (e–f) in the studies reviewed (*n* = 52) in an examination of the peer‐reviewed literature applying Q to conservation issues: (a) number of respondents (sample size), (b) number of items in the concourse, (c) number of items to sort, (d) ratio of items to respondents, (e) percentage of studies that used each method for factor extraction (^*^), only factor analysis was indicated in the study without specifying centroid or principal components analysis, (f) number of factors interpreted, (g) percentage of studies that used each rotation method, and (h) percentage of the variance explained by all factors.

The number of respondents typically ranged from 26 to 46 (Fig. 3). A few studies administered Q to exceptionally large samples beyond 100 individuals (e.g., Milcu et al. 2014; Carmenta et al. 2017). The time required for research design (developing the concourse and Q‐set) was typically 2–4 months (*n* = 7). There were exceptions of a few weeks (Chamberlain et al. 2012) and up to a few years (Ockwell 2008). Collecting each Q‐sort took 30–90 min (if face to face; *n* = 4). Based on our own experience, a sensible estimation of the face to face administration time for each respondent is 1 h plus the time to approach (traveling time and introducing the study). This may need adjustment depending on the number and length of items to rank and on respondents’ reading level.

The Q methodology has been used in conservation contexts to understand people's subjectivity broadly, such as their attitudes, beliefs, values, or perceptions about a subject matter. These include studies exploring a variety of issues such as multifunctional forestry (e.g., Nijnik et al. 2010) or invasive crab management (Falk‐Petersen 2014). In a few cases, Q has been applied to understand more abstract concepts, such as management styles (Brodt et al. 2004) or schools of thought (Neff 2014).

The perspectives found are not necessarily in opposition to one another. Rather, perspectives reveal different ways of “doing” or different ways of “seeing” among individuals or groups. These applications include choices for landscape management (Milcu et al. 2014), farming styles (e.g., Davies & Hodge 2007), or options for designing an aquarium experience (Sickler et al. 2006).

We categorized Q applications in conservation into four themes: ascertaining management options (*n* = 20), critical reflection (*n* = 16), policy appraisal and acceptability (*n* = 10), and addressing conflict (*n* = 6). We assigned a single category to each study. If the study covered more than one category, we chose the predominant one.

### Management Alternatives

One common use of Q is to elicit alternatives or solutions to conservation problems. These uses are at the stage of policy design, when measures have not yet been implemented and researchers aim to understand various options. For example, understanding the views of those who may be affected by a policy can help managers choose instruments adapted to each motivation (Nordhagen et al. 2017; Zabala et al. 2017).

Identifying views about management alternatives is also useful to understand power dynamics among decision makers. For example, in a study regarding the management of a protected area (Mattson et al. 2011), the management approaches identified using Q anticipated changes in the governing board. A few months after the study, the views of individuals who entered the board had aligned with the view predominating among other members of the board, whereas the only board member whose view was distinct according to the study resigned.

### Critical Reflection

Because conservation is a value‐laden discipline, Q has been applied to critically reflect on the values that underpin the activity of conservation professionals and researchers (e.g., Sandbrook et al. 2011; Blanchard et al. 2016). Debates studied with Q include diverse perspectives on the value of market‐based policies in conservation (Holmes et al. 2016); interpretations of broad, contested, or ill‐defined concepts, such as ecosystem services (Fisher & Brown 2014; Bredin et al. 2015*a*); and the role of science in conservation (Bischof 2010).

In explicating perspectives that underlie conservation practice and research, Q informs understanding of how the relationship between facts and values is perceived by different groups of people and helps in the development of self‐awareness. These reflections are useful, for example, to clarify discourses about how policies should be shaped or to challenge prevailing assumptions about the existence of strongly positive or negative stances. As an example of the latter, Sandbrook et al. (2013) in their study of conservationist's views on market‐based approaches to conservation, found that few embrace or discard these instruments completely and that the main difference was in the emphasis given to their potential versus that given to their caveats.

### Policy Appraisal

The Q methodology has been used to appraise current or prospective conservation policy and to explore whether and why a policy mechanism is or will be accepted. Example applications include the acceptability of wildlife‐monitoring systems (Chandran et al. 2015), of marine protected areas (Gall & Rodwell 2016), and of proposed land‐use changes (Swaffield & Fairweather 1996). Unexpected perceptions about proposed policies can be uncovered. For example, a study on perceived potential measures for fire management found that from four main possible approaches identified among respondents, only two were reflected in existing policy proposals (Ockwell 2008).

### Conflict Resolution

Addressing conflict is a common endeavor in conservation, and Q is particularly useful for such a purpose. It has been used mainly in conflicts related to the management of large terrestrial wildlife (Mazur & Asah 2013; Bredin et al. 2015*b*). In these controversies, stakeholders disagree about the way forward, tension is high, and further scientific evidence may be insufficient to overcome frictions.

When a situation is intractable or gridlocked (Mazur & Asah 2013), Q helps researchers identify which stakeholders have the most conflicting views and “crystallize points of contention” (MacDonald et al. 2015), allowing actors with contested opinions to disagree openly and to concretize their arguments (Chandran et al. 2015). A more precise understanding of the common ground and of disagreements can be instrumental to mediate conflicts and find solutions. Also, Q can uncover new unexpected connections among topics, provide common vocabulary (MacDonald et al. 2015) that facilitates dialogue, and identify unexpected similarities in the views of seemingly opposing stakeholders.

## Discussion

The Q methodology facilitates understanding of plural perspectives through a well‐defined procedure that is appropriate for use with a relatively small sample of respondents. The structured nature of the process makes it relatively easy to follow, although those considering its use must recognize that Q is not a purely quantitative or positivist methodology, and that the interpretation of the results is necessarily and appropriately subjective to some extent. We believe Q is potentially an important tool for researchers and practitioners who wish to understand complex viewpoints among stakeholders.

Understanding perspectives allows researchers to acknowledge views that are marginalized (Ockwell 2008) or little known. The Q methodology can also unveil latent or hidden views (Mazur & Asah 2013) that do not necessarily emerge through other methods because these views are controversial and respondents are not vocal about them or do not articulate them explicitly otherwise. It can also facilitate participation (Ockwell 2008) and deliberation (Mazur & Asah 2013) in decision‐making processes.

Based on our review of Q applications in conservation, future Q studies would benefit from clearly recording each step of the process to justify and report, where possible, the key research decisions sufficiently, namely: the selection of items for sorting, the sample of respondents, and the main analytical decisions (listed above). If working beyond the typical Q procedure (Fig. 1), it is important to be explicit about what is different in the given study and why, for readers to understand when such variations are useful. For reporting, we suggest justifying the choice of Q in contrast to other plausible approaches and using the elements in Fig. 1 as a checklist.

A Q study may be developed further either with less conventional study designs or by combining it with other approaches. For example, photographs may be used instead of statements (e.g., to investigate landscape‐related issues [Milcu et al. 2014]). Two aspects of the same issue can be analyzed by asking the same respondents to rank two different sets of items (e.g., problems and solutions [Carmenta et al. 2017]). Respondents can also rank items with different conditions of instruction, for example, according to what they believe represents an organization's view instead of their own (Clare et al. 2013). This can uncover divergences between individuals’ opinions and those of the institutions they work for, which may be relevant in major conservation organizations. Another option is to design a survey based on Q results to estimate the frequency of perspectives in a wider population (Danielson 2009). Because data are collected in a cross‐sectional manner (with few exceptions), in order to understand temporal dynamics of perspectives studies need more complexity than that outlined here. In one of the few examples of such a complex design, Davies and Hodge (2007, 2012) found how some perspectives are more permanent than others and that they are relatively fluid.

With increasing awareness of the importance of the human factor in conservation research and practice, integrating and understanding diverse opinions has become a pressing need. Among several social science methodologies that are available for such research goals, Q stands out for its ability to identify the range of perspectives in a structured way and for the nuanced positions that emerge from asking respondents to make relative choices between items. If used appropriately, Q has considerable potential to help identify areas of consensus and disagreement around key conservation topics, which can then be used to resolve conflicts, assess management alternatives, appraise policies, or facilitate critical reflection.

## Supporting information

The literature review search string (Appendix S1), a list of articles in the review (Appendix S2), and information extracted from each study for the review (Appendix S3) are available online. The authors are solely responsible for the content and functionality of these materials. Queries (other than absence of the material) should be directed to the corresponding author
