# Supplementary material for: When and how to use Q methodology to understand perspectives in conservation research
Source: Conserv Biol. 2018 Jul 20;32(5):1185–94. doi: 10.1111/cobi.13123 (PMC6849601; doi:10.1111/cobi.13123)
Supplement: Supplementary file 1 — The literature review search string (Appendix S1), a list of articles in the review (Appendix S2), and information extracted from each study for the review (Appendix S3) are available online. The authors are solely responsible for the content and functionality of these materials. Queries (other than absence of the material) should be directed to the corresponding author [file COBI-32-1185-s001.doc]

# Appendices

## S1. Approach to sample Q studies in conservation

We sampled studies using keyword search in Scopus, covering biodiversity or conservation, during all years available (on May 2016; see below for the exact search string). This sampling resulted in 204 articles and 8 review articles (none of the latter were actually on Q). A screening of titles and abstracts reduced the sample to 38 articles. Next, we added 14 further studies based on replicated searches until March 2017 and references of the initial sample. Finally, we searched in Web of Science and in the archives of ‘Operant Subjectivity’ (a journal specialized in Q but not indexed), although these searches did not yield further relevant studies.

(TITLE-ABS-KEY (q method*) AND TITLE-ABS-KEY (biodiver*) OR TITLE-ABS-KEY (conserv*)) AND DOCTYPE (ar OR re OR ip) AND SUBJAREA (mult OR agri OR bioc OR immu OR neur OR phar OR mult OR medi OR nurs OR vete OR dent OR heal OR mult OR arts OR busi OR deci OR econ OR psyc OR soci) AND (EXCLUDE(SUBJAREA ,"BIOC")) AND (EXCLUDE (SUBJAREA ,"MEDI"))

From the articles selected, one article was excluded (Rutherford et al. 2009) because the case was already reported in another study (Chamberlain et al. 2012).

## S2. List of articles in the review

|  | **Authors** | **Year** | **Title** | **Journal** |
| --- | --- | --- | --- | --- |
| 1 | Benitez-Capistros, Hugé, Dahdouh-Guebas, Koedam | 2016 | Exploring conservation discourses in the Galapagos Islands: A case study of the Galapagos giant tortoises | Ambio 45, 706–724. |
| 2 | Berry, Fabok, Blicharska, Bredin, Llorente, Kovacs, Geamana, Stanciu, Termansen, Jääskeläinen, Haslett, Harrison | 2016 | Why conserve biodiversity? A multi-national exploration of stakeholders’ views on the arguments for biodiversity conservation | Biodiversity and Conservation |
| 3 | Bischof | 2010 | Negotiating uncertainty: Framing attitudes, prioritizing issues, and finding consensus in the coral reef environment management ‘crisis’ | Ocean and Coastal Management, 53, 597–614. |
| 4 | Blanchard, Sandbrook, Fisher, Vira, | 2016 | Investigating consistency of a pro-market perspective amongst conservationists | Conservation and Society 14, 112 |
| 5 | Bredin, Lindhjem, van Dijk, Linnell | 2015 | Mapping value plurality towards ecosystem services in the case of Norwegian wildlife management: A Q analysis | Ecological Economics, 118, 198–206. |
| 6 | Bredin, Linnell, Silveira, Tôrres, Jácomo, Swenson | 2015 | Institutional stakeholders’ views on jaguar conservation issues in central Brazil | Global Ecology and Conservation, 3, 814–823. |
| 7 | Brodt, Klonsky, Tourte, Duncan, Hendricks, Ohmart, Verdegaal | 2004 | Influence of farm management style on adoption of biologically integrated farming practices in California | Renewable Agriculture and Food Systems, 19, 237–247. |
| 8 | Cairns | 2012 | Understanding science in conservation: A q method approach on the Galápagos islands | Conservation and Society, 10, 217–231. |
| 9 | Cavanagh, Hill, Knowland, Grant | 2016 | Stakeholder perspectives on ecosystem-based management of the Antarctic krill fishery | Marine Policy, 68, 205–211. |
| 10 | Chamberlain, Rutherford, Gibeau | 2012 | Human Perspectives and Conservation of Grizzly Bears in Banff National Park, Canada | Conservation Biology, 26, 420–431. |
| 11 | Chandran, Hoppe, De Vries, Georgiadou | 2015 | Conflicting policy beliefs and informational complexities in designing a transboundary enforcement monitoring system | Journal of Cleaner Production, 105, 447–460. |
| 12 | Clare, Krogman, Caine | 2013 | The balance discourse: A case study of power and wetland management | Geoforum, 49, 40–49. |
| 13 | Clarke | 2002 | Understanding sustainable development in the context of other emergent environmental perspectives | Policy Sciences, 35, 69–90. |
| 14 | Davies, Hodge | 2007 | Exploring environmental perspectives in lowland agriculture: A Q methodology study in East Anglia, UK | Ecological Economics, 61, 323–333. |
| 15 | Davies, Hodge | 2012 | Shifting environmental perspectives in agriculture: Repeated Q analysis and the stability of preference structures | Ecological Economics, 83, 51–57. |
| 16 | Falk-Petersen | 2014 | Alien invasive species management: Stakeholder perceptions of the barents sea king crab | Environmental Values, 23, 701–725. |
| 17 | Fisher, Brown | 2014 | Ecosystem services concepts and approaches in conservation: Just a rhetorical tool? | Ecological Economics, 108, 257–265. |
| 18 | Gall, Rodwell | 2016 | Evaluating the social acceptability of Marine Protected Areas | Marine Policy, 65, 30–38. |
| 19 | Gruber | 2011 | Perspectives of effective and sustainable community-based natural resource management: An application of Q methodology to forest projects | Conservation and Society, 9, 159. |
| 20 | Hagan, Williams | 2016 | Oceans of Discourses: Utilizing Q Methodology for Analyzing Perceptions on Marine Biodiversity Conservation in the Kogelberg Biosphere Reserve, South Africa | Frontiers in Marine Science 3 |
| 21 | Hamadou, Moula, Siddo, Issa, Marichatou, Leroy, Antoine-Moussiaux | 2016 | Mapping stakeholder viewpoints in biodiversity management: an application in Niger using Q methodology | Biodiversity and Conservation, 25, 1973–1986. |
| 22 | Holmes, Sandbrook, Fisher | 2016 | Understanding conservationists’ perspectives on the new conservation debate | Conservation Biology. |
| 23 | Hugé, Vande Velde | 2016 | Mapping discourses using Q methodology in Matang Mangrove Forest, Malaysia | Journal of Environmental Management, 1–10. |
| 24 | Jacobsen, Linnell | 2016 | Perceptions of environmental justice and the conflict surrounding large carnivore management in Norway — Implications for conflict management | Biological Conservation 203, 197–206 |
| 25 | Kamal, Grodzinska-Jurczak | 2014 | Should conservation of biodiversity involve private land? A Q methodological study in Poland to assess stakeholders’ attitude | Biodiversity and Conservation, 23, 2689–2704. |
| 26 | Kamal, Kocór, Grodzińska-Jurczak | 2014 | Quantifying human subjectivity using Q method: When quality meets quantity | Qualitative Sociology Review, 10, 60–79. |
| 27 | Kindermann, Gormally | 2013 | Stakeholder perceptions of recreational and management impacts on protected coastal dune systems: A comparison of three European countries | Land Use Policy, 31, 472–485. |
| 28 | MacDonald, Murray, Patterson | 2015 | Considering social values in the seafood sector using the Q-method | Marine Policy, 52, 68–76. |
| 29 | Mattson, Byrd, Rutherford, Brown, Clark | 2006 | Finding common ground in large carnivore conservation: mapping contending perspectives | Environmental Science and Policy, 9, 392–405. |
| 30 | Mattson, Clark, Byrd, Brown, Robinson | 2011 | Leaders’ perspectives in the Yellowstone to Yukon Conservation Initiative | Policy Sciences, 44, 103–133. |
| 31 | Mazur, Asah | 2013 | Clarifying standpoints in the gray wolf recovery conflict: Procuring management and policy forethought | Biological Conservation, 167, 79–89. |
| 32 | Milcu, Sherren, Hanspach, Abson, Fischer | 2014 | Navigating conflicting landscape aspirations: Application of a photo-based Q-method in Transylvania, Central Romania | Land Use Policy, 41, 408–422. |
| 33 | Neff | 2014 | Research Prioritization and the Potential Pitfall of Path Dependencies in Coral Reef Science | Minerva, 52, 213–235. |
| 34 | Neff | 2011 | What research should be done and why? Four competing visions among ecologists | Frontiers in Ecology and the Environment, 9, 462–469. |
| 35 | Neff, Larson | 2014 | Scientists, managers, and assisted colonization: Four contrasting perspectives entangle science and policy | Biological Conservation, 172, 1–7. |
| 36 | Nijnik, Nijnik, Lundin, Staszewski, Postolache | 2010 | A study of stakeholders’ perspectives on multi-functional forests in Europe | Forests, Trees and Livelihoods, 19, 341–358. |
| 37 | Nordhagen, Pascual, Drucker | 2017 | Feeding the Household, Growing the Business, or Just Showing Off? Farmers’ Motivations for Crop Diversity Choices in Papua New Guinea | Ecological Economics, 137, 99–109 |
| 38 | O’Riordan, McDonagh, Mahon | 2016 | Local knowledge and environmentality in legitimacy discourses on Irish peatlands regulation | Land use policy 59, 423–433 |
| 39 | Ockwell | 2008 | ‘Opening up’ policy to reflexive appraisal: A role for Q Methodology? A case study of fire management in Cape York, Australia | Policy Sciences, 41, 263–292. |
| 40 | Pereira, Fairweather, Woodford, Nuthall | 2016 | Assessing the diversity of values and goals amongst Brazilian commercial-scale progressive beef farmers using Q-methodology | Agricultural Systems, 144, 1–8. |
| 4 | Rastogi, Hickey, Badola, Hussain | 2013 | Diverging viewpoints on tiger conservation: A Q-method study and survey of conservation professionals in India | Biological Conservation, 161, 182–192. |
| 42 | Rodríguez-Piñeros, Mayett-Moreno | 2014 | Forest owners’ perceptions of ecotourism: Integrating community values and forest conservation | Ambio, 99–109. |
| 43 | Rust | 2017 | Can stakeholders agree on how to reduce human–carnivore conflict on Namibian livestock farms? A novel Q-methodology and Delphi exercise | Oryx, 51(2), 339–346 |
| 44 | Sandbrook, Fisher, Vira | 2013 | What do conservationists think about markets? | Geoforum, 50, 232–240. |
| 45 | Sandbrook, Scales, Vira, Adams | 2011 | Value plurality among conservation professionals | Conservation Biology, 25, 285–94. |
| 46 | Sickler, Fraser, Webler, Reiss, Boyle, Lyn, Lemcke, Gruber | 2006 | Social Narratives Surrounding Dolphins: Q Method Study | Society & Animals, 14, 351–382. |
| 47 | Swaffield, Fairweather | 1996 | Investigation of attitudes towards the effects of land use change using image editing and Q sort method | Landscape and Urban Planning, 35, 213–230. |
| 48 | Urquhart, Courtney, Slee | 2012 | Private woodland owners’ perspectives on multifunctionality in english woodlands | Journal of Rural Studies, 28, 95–106. |
| 49 | Visser, Moran, Regan, Gormally, Skeffington | 2007 | The Irish agri-environment: How turlough users and non-users view converging EU agendas of Natura 2000 and CAP | Land Use Policy, 24, 362–373. |
| 50 | West, Cairns, Schultz | 2016 | What constitutes a successful biodiversity corridor? A Q-study in the Cape Floristic Region, South Africa | Biological Conservation, 198, 183–192. |
| 51 | Winkler, Nicholas | 2016 | More than wine: Cultural ecosystem services in vineyard landscapes in England and California | Ecological Economics, 124, 86–98. |
| 52 | Zabala, Pascual, Garcia-Barrios | 2017 | Payments for pioneers? Revisiting the role of external rewards for sustainable innovation under heterogeneous motivations | Ecological Economics, 135, 234–245 |

## S3. Information extracted from each study for the review

From each of the articles, we extracted the following information:

- Question/purpose,
- Result/output (primarily the names of the factors or perspectives),
- Method of administration,
- Decision context,
- Geographical scale,
- Advantages,
- Disadvantages,
- Recommendations,
- Use with other methods,
- Sample size,
- Sampling method,
- Sampling stratification,
- Duration of the research process.

We also searched for information about differences and commonalities across perspectives. A series of additional variables specific to Q were also included in the database. The Q-specific variables are:

- Q-sample stimuli,
- Concourse sources and size,
- Q-set size,
- Methods for extraction, rotation and flagging,
- Number of factors,
- Factor labels,
- Criteria used to justify the number of factors extracted,
- Variability explained by the factors,
- Sampling method,
- Condition of instruction.

Very few studies justify why they chose Q or contrasted it with other methods (although many studies justify the research choices within the methodology). Rather, studies directly explain what the methodology is for and what are its advantages according to standard literature (e.g. Brown 1980, Watts & Stenner 2012). Therefore we annotated the rationale only for studies which explicitly stated why they chose the methodology and/or that contrast it with other methodologies that could have been chosen. Likewise, most studies mention at least a few advantages of the methodology, but these are based on direct references, they tend to be homogeneous and part of the explanation of the methodology at the beginning of a methods section. For that reason, ‘advantages’ and ‘disadvantages’ were annotated only if they stemmed directly from the experience of the author(s) in the given study.
